# Supplementary material for: Are carbon emissions trading and green financial instruments synergistic? -Comprehensive quantitative research based on content analysis
Source: PLoS One. 2024 Mar 7;19(3):e0298601. doi: 10.1371/journal.pone.0298601 (PMC10919601; doi:10.1371/journal.pone.0298601)
Supplement: S1 Data — (ZIP) [file pone.0298601.s001.zip › Data/地级市Z_F工作报告.zip!/╔┬╬≈/╬╝─╧╩╨/└┤╫╘╣1⁄2╓┌║┼í╛┬φ┐╦╩2╛▌═°í┐_2016.docx]

各位代表：

　　现在，我代表市人民政府向大会作工作报告，请予审议，并请市政协委员及列席人员提出意见。

　　一、过去六年工作回顾

　　本届政府任期六年来，在省委、省政府和市委的正确领导下，紧扣科学发展、富民强市主题，聚焦转变发展方式主线，加快推进城区、园区、景区融合发展，新型工业化、城镇化、信息化、农业现代化同步建设，开拓创新，埋头苦干，全市经济社会持续较快发展，较好地完成了市四届人大确定的各项目标任务。

　　经济持续快速增长，综合实力迈上新台阶。全市经济总量跨越千亿元大关，2015年达到1469.08亿元，是2009年的2.3倍，年均增长12.6%。人均GDP由11728元提高到27452元，年均增长12.4%。地方财政收入完成72.06亿元，是2009年的2.55倍，年均增长16.9%。社会消费品零售总额完成503.22亿元，是2009年的2.5倍，年均增长16.5%。城乡居民收入分别达到25472元和8705元，年均增长13.4%、17.4%。主要经济指标增幅均超过全省平均水平，经济发展的质量和效益明显提升。

　　基础设施全面改善，支撑能力不断增强。围绕制约发展的瓶颈问题，创新投融资方式，建成了一批打基础、利长远的重大项目。累计招引项目1993个，到位资金2675.5亿元，年均增长22%。实施重点项目898个，完成投资5700亿元。全社会固定资产投资累计完成8655.2亿元，年均增长26.5%。大西高铁建成运营，黄韩侯铁路全线贯通，渭蒲高速、渭玉高速、西潼高速改扩建和渭南至大荔一级公路等20个国省道升等改造项目建成通车。渭河综合整治主体项目基本完成，整体防洪能力大幅提升。抽黄供水工程输水管道全面建成，引石济白建成受益，恢复改造农田灌溉面积130万亩，农村自来水入户率达到90%以上。新增电力装机202万千瓦，供电保障能力超过400万千伏安。“气化渭南”稳步推进，县城以上气化率达到50%以上，加快发展的基础支撑越来越坚实。

　　发展方式持续优化，产业转型升级取得突破。加速推进新型工业化，传统工业加快升级，龙钢、金钼、陕化扩能技改全面完成，蒲城清洁能源、秦电7#8#机组、华县10万吨丁二醇、富平生态水泥、青啤搬迁等重大工业项目建成投产，能源化工等主导产业加速向中高端延伸。战略性新兴产业快速发展，3D打印、新能源汽车成为发展新亮点。渭南高新区晋升国家级高新区，省级开发区达到5个，开发区工业园区发展到20个。产值过10亿元企业由16户增加到31户，陕西黑猫焦化和伟恒生物、红星美羚等3户企业成功上市，新增省级名牌产品67个，中国驰名商标达到8个。规模以上工业总产值、增加值完成1975.94亿元和610.5亿元，分别是2009年的2.8倍和2.5倍。现代农业快速发展，粮食生产实现 “十二连丰”，水果、蔬菜产量达到310.6万吨和253.2万吨，分别是2009年的1.3倍和1.5倍，畜牧业产值达到82.65亿元，较2009年翻了一番多。现代农业园区发展到346个，市级以上龙头企业发展到388家，白水苹果、大荔冬枣、蒲城酥梨、临渭葡萄、合阳红提跻身“中国果品区域公用品牌50强”，韩城大红袍花椒、华县大葱等12个农产品通过国家地理标志认证。第三产业全面提速，华山成功晋升5A级景区，3A级以上景区发展到33家，大华山旅游格局初步形成，2015年接待游客4219万人次、旅游综合收入323.8亿元，分别是2009年的3.8倍和6.3倍。电子商务、现代物流、健康养老等新业态加速发展，蒲城、白水成功创建省级电子商务示范县，新增限额以上贸易企业624户。一、二、三产结构调整为14.6∶50.2∶35.2，工业主导地位更加突出，三次产业协调发展格局全面形成。

　　新型城镇化加速推进，城乡面貌深刻变化。秦东城镇群建设格局初步形成，华县成功撤县设区。中心城市“一河两岸、五大组团”竞相发展，城市功能更加完善、品位大幅提升。市民综合服务中心、文化艺术中心、体育中心、博物馆、万达广场等城市地标相继建成投用，渭河湿地公园、湭河公园等成为市民休闲健身的新亮点。新修改造道路230余公里，“断头路”基本消除，网格状城市道路系统基本形成，城区三座大桥收费站全部撤销，西潼高速渭南西入口改扩建建成通车、东入口改扩建启动实施。城市自来水普及率、污水处理率和气化率分别达到97%、98.4%和87%，城市绿化覆盖率达到38%，国家卫生城市和省级园林城市成功创建。县城扩容提质成效明显，国家级卫生县城达到5个，省级卫生县城实现全覆盖，合阳成功创建国家园林县城。22个中省市重点镇和文化旅游名镇建设加速推进，特色日益凸显。建成城市社区168个、农村社区971个，61.4万农民进城落户，城镇化率达到41.8%。

　　生态治理不断加强，绿色发展优势逐步彰显。突出节能减排、重点区域整治和造林绿化，努力将生态建设向纵深推进。淘汰小水泥、小钢铁等落后产能670.7万吨，单位GDP能耗累计下降21%，主要污染物减排如期达标。渭河治污成效明显，出境断面水质由劣Ⅴ类提升到Ⅳ类。“治污降霾·保卫蓝天”行动扎实有效，累计拆除燃煤锅炉1325台，淘汰黄标车老旧车4.1万辆，在全省率先引进纯电动公交车84辆，2015年中心城市空气质量优良天数达到263天。秦岭北麓生态保护全面实施，采取铁腕措施整治非法采石和土法石灰窑。秦东大地园林化深入推进，新增绿地262.6万亩，治理水土流失2743平方公里，建成国家湿地公园8个，森林覆盖率由18.9%提高到25.5%。210个美丽乡村示范村建设普遍达标，大荔、合阳成为全国美丽乡村示范县，8镇12村被评为省级生态镇村，“大水、大绿、大空间”的生态格局逐步展现。

　　民生改善力度加大，人民群众得到更多实惠。坚持把增进人民福祉作为一切工作的出发点和落脚点，财政民生累计投入1233亿元，占到财政支出的82.3%。新增就业43.2万人，城镇登记失业率控制在3.2%以内。新建改建公办幼儿园498所，学前教育全面普及，创建标准化学校373所，6个县市区通过省级“双高双普”验收，职业教育和民办教育蓬勃发展。市中心医院建成投用，县级公立医院改革稳步推进，二级甲等以上医院发展到22家，城乡居民大病保险全面实施，新农合参合率达99%以上。农村五保供养标准提高2.5倍，城乡低保应保尽保。11.6万户城乡低收入群众通过安居工程、扶贫搬迁住进新居，80.5万人实现脱贫。51万群众享受移民后期扶持政策，216.6万群众饮水安全问题得到解决。国家公共文化服务体系示范区创建深入推进，我市被确定为国家体育产业联系点城市，群众文体生活丰富多彩，华阴老腔、韩城行鼓荣登猴年央视春晚。国家双拥模范城“五连冠”创建通过省级验收。平安渭南、法治渭南建设扎实推进，安全生产形势平稳，社会大局和谐稳定。

　　改革创新深入推进，对外开放逐步扩大。坚持把改革创新作为加快发展的不竭动力，食品药品监管体制改革、文化惠民“一元剧场”、农技服务“大荔模式”、财政惠民“一卡通”成为全国改革典范，商事制度、公务用车制度改革走在全省前列。韩城计划单列市试点、国家中小城市综合改革试点进展顺利。政府机构和事业单位改革稳步推进，市政府机构精简至48个。简政放权深入推进，下放取消市级管理权限1303项，对外公开市级部门“权力清单”，全面推行“并联审批、一厅式办公”，群众办事更加便捷。镇村综合改革基本完成，农村土地确权登记颁证、集体林权制度改革稳步推进。组建城投、产投、华旅、农投等市属国有公司，混合所有制经济加快发展。与16家高校院所签订战略合作协议，引进高层次人才827名，组建工程技术研究中心38个，建成“众创空间”20个，全社会研发投入占GDP比重突破1%。晋陕豫黄河金三角区域合作上升为国家战略，西渭融合发展稳步推进，渭南海关和出入境检验检疫局开关开检，累计利用外资2.1亿美元、进出口贸易额达到12亿美元。

　　政府自身建设得到加强，执政能力进一步提升。扎实开展党的群众路线教育实践活动、“三严三实”专题教育，持续开展纪律作风大整顿大排查，严格执行中央八项规定，加强审计监督和效能监察，财政预决算全面公开，“三公”经费大幅下降。深入推进党风廉政建设和反腐败工作，坚决纠正“四风”，先后查处违纪违法公职人员2524人，干部作风持续改进。完善重大事项依法决策制度，严格按照法定权限和程序履行职责，加强行政执法监督，依法行政示范市创建工作受到省政府通报表彰。自觉接受人大法律监督和政协民主监督，人大议案、政协提案办结率连年达到100%。全面推进政务公开，百姓问政、向人民报告、邀请公民进政府等活动受到群众好评，人民网留言办理连续五年被评为全国先进，政府公信力和工作效能明显提升。

　　同时，国防动员、国家安全、人民防空、民族宗教、外事侨务不断加强，妇女儿童、老龄、残疾人事业全面进步，统计、地震、气象、档案、地方志等工作都取得了新的成绩。

　　各位代表，过去的六年，是我市经济社会平稳较快发展、城乡面貌变化巨大、人民群众得到较多实惠的六年。六年来成绩的取得，是市委正确领导的结果，是市人大、市政协和社会各界鼎力支持的结果，是560万渭南人民共同奋斗的结果。在此，我谨代表市人民政府，向各位代表、各位委员，向奋战在各条战线上的广大干部群众，向长期以来关心支持渭南发展的各界人士、驻渭部队和武警官兵，表示衷心的感谢和崇高的敬意！

　　各位代表，回顾过去六年的工作，成绩令人振奋，经验弥足珍贵，六年的发展实践使我们深深地体会到：必须把加快发展作为强市之要，努力保持中高速增长，不断做大经济总量，持续转变发展方式，加速向中高端水平迈进；必须把改革创新作为动力之源，坚持以改革破难题、添活力，大力实施创新驱动战略，积极扩大对外开放，持续增强发展后劲；必须把生态文明建设作为长远之策，坚守绿水青山，坚持不懈地建设美丽渭南；必须把改善民生作为执政之本，脚踏实地为群众办实事、做好事、解难事，真正做到发展为了人民、发展依靠人民、发展成果由人民共享；必须把强化责任担当作为关键之举，持续转变作风，干在实处，走在前列，努力创造经得起实践、人民和历史检验的实绩。

　　在充分肯定成绩的同时，我们也清醒认识到发展面临的一些突出矛盾和问题：经济总量依然偏小、结构还不优，产业转型升级任务繁重而紧迫；城镇化水平依然较低，城乡协调发展差距明显；民生改善、扶贫攻坚、生态建设等方面与群众期盼还有很大差距；少数政府工作人员作风和能力还不能完全适应全面从严治党、建设服务型政府的要求；受经济下行等因素影响，个别指标没有达到预期目标，等等。对此，我们一定高度重视，下大气力加以解决。

　　二、今后五年的基本思路和目标任务

　　未来五年，是新一届政府任期的五年，也是“十三五”规划全面实施的五年。综合研判这一时期，虽然我们面临着经济下行压力加大、结构调整阵痛、资源环境约束趋紧等诸多挑战，但也面临着很多有利因素和机遇：国家深入推进“一带一路”建设，大力实施创新驱动战略，推进区域协调发展，加快培育发展新动能，我们将获得更多政策支持；省上加快推进西渭一体化发展，持续推进陕西东大门建设，为我们补齐短板提供了有力支撑；经过多年的努力，我们积聚了发展的坚实基础和巨大潜能，正处在转型发展的新阶段，全市干部群众谋求新发展、推动新跨越的氛围空前浓厚，为实现追赶超越提供了强大动力。只要我们顺应民心、凝聚民力，把握机遇、主动作为，更加扎实地做好各项工作，就一定能够夺取全面建成小康社会决战的伟大胜利。

　　今后五年全市经济社会发展的基本思路和主要目标是：全面贯彻党的十八大和十八届三中、四中、五中全会精神及习近平总书记系列重要讲话精神，坚持“四个全面”战略布局和创新、协调、绿色、开放、共享发展理念，以加快发展、追赶超越、决胜小康为主题，大力实施创新驱动、项目带动、产业提升、板块引领战略，联动推进新型城镇化、新型工业化、信息化和农业现代化融合发展，在提高质量和效益的基础上，经济增长高于全省平均水平，进入全省发展第一方阵。到2020年，力争生产总值达到2500亿元、城乡居民收入达到45900元和16000元，较2010年实现“三个翻一番多”，现行标准下农村贫困人口全部脱贫，城乡一体化全面推进，城镇化率达到60%，生态环境与经济发展深度融合，人民生活水平和质量进一步提高，同步够格全面建成小康社会，富裕、和谐、文明、绿色、开放新渭南建设迈上更高水平。

　　今后五年政府工作的重点任务：

　　一是加快产业转型升级，着力构筑现代产业体系。推进一、二、三产协调发展，着力构筑产业发达、结构合理、优势明显的现代产业体系。坚持工业强基战略，以高端化、规模化、品牌化为导向，以高新区、经开区、卤阳湖和县域特色产业园区为载体，培育壮大清洁能源化工、装备制造、食品医药、冶金绿色建材、新能源汽车、三维制造和光电等六大优势产业集群，打造西部绿色能源化工和先进制造业聚集区。力争到2020年，工业总产值达到4000亿元。持续推进现代农业强市建设，以现代农业园区为引领，建设400万亩水果、200万亩瓜菜、千万头生猪、百万只奶山羊、20万头奶牛产业示范基地，大力发展涉农加工，建成全省绿色食品基地和现代畜牧业示范区，打造国家现代农业示范区。加快发展现代服务业，整合山水城陵等资源，以华山为龙头，推进全域旅游，争创4A级以上景区13个，建设2个国家旅游度假区，打造国际山水文化旅游目的地。推动现代物流、金融、科技信息和商贸服务深度融合，打造通航物流港、中西部重要的物流集散地和现代服务业聚集区。

　　二是强化基础设施建设，着力夯实发展基础。加快补短板、提标准，不断强化基础设施支撑能力。积极构建现代综合交通网络体系。建设韩城、华山支线机场；推进渭南至蒲城、富平至韩城、临潼至潼关城际铁路和中心城市轨道交通网建设；实施合凤、榆商高速和京昆高速改扩建，构建“两纵四横”高速枢纽网络，实施国道310、省道209等公路升等改造，形成“两纵两横”一级公路主骨架，新建改建一二级公路500公里，实现城区、景区、园区间连通二级以上公路全覆盖，新建合阳至临猗、大荔至永济两座黄河大桥，推进晋陕豫省级互联互通。加快建设渭南水系。按照利用客水、蓄住洪水的治水思路，实施重点水源、防洪安澜、城乡供水、灌溉排水、水保生态、水面景观六大工程，建成水兴渭南、水润秦东的安全健康优美水系。实施渭南和富平热电联产、渭北煤电一体化、智能电网、“气化渭南”二期工程等重点项目，提升能源综合保障能力。加强信息网络建设，推进大数据应用示范工程，建设“智慧渭南”。

　　三是加快城乡协调发展，着力提升城镇带动能力。坚持以统筹城乡发展、优化产业布局为导向，突出地域特色和以人为核心，加快构建“一核三带五板块”产业与城乡互动融合发展的秦东新型城镇群，打造关中城镇群副中心城市。做大中心城市规模，加快“一河两岸、五大组团”发展，推进渭华城市带建设，到2020年建成百万人口中心城市。加快韩城黄河金三角区域中心城市建设，推进富阎一体化，将富平打造成市域经济发展新的增长极。推进蒲城、大荔撤县设市，做强澄城、华阴、合阳、白水、潼关等县域中心城市。推动中心城市高端制造和现代服务业、韩城新型工业和循环经济、富蒲航空和卤阳湖湿地、大华山山水文化旅游、蒲白澄合载能工业和绿色生态等五大板块产城融合发展，推进富平—韩城、渭南—潼关和黄河西岸城乡统筹示范带建设，全面加快中省市重点示范镇和文化旅游名镇建设，建成300个标准化新型农村社区，实现30%的农村社区化，120万人进城落户。

　　四是实施创新驱动战略，着力增强发展的动力和活力。牢固树立创新发展意识，以推进国家中小城市综合改革试点为契机，全力构建创新驱动发展格局，持续深化行政管理、投融资、财税金融、现代市场体系建设等重点领域的体制机制改革，打造国家综合改革创新示范区。持续推进创新型渭南建设，强化企业创新主体地位，深入推进校地校企合作，建立校企创新联盟，促进科技成果承接转化，引领产业转型升级。力争建成省级以上技术中心29家、检验检测中心8家。加快创新服务型政府建设，营造良好环境，推进大众创业、万众创新，激发全社会创业创新激情。推动发展空间创新，积极参与“一带一路”建设，加强与沿线城市的交流合作，深入推进晋陕豫黄河金三角区域合作和西渭融合发展，大力发展开放型经济。

　　五是加强生态文明建设，着力厚植绿色发展优势。牢固树立尊重自然、顺应自然、保护自然的生态文明理念，坚定不移走绿色发展之路。实施主体功能区发展战略，加快黄河、渭河、洛河及秦岭北麓、渭北丘陵沟壑五大生态区建设，围绕南北山地森林化、南北台塬绿色化、中部平原园林化，推进黄渭洛三河沿岸防护林、国省干线公路绿化工程以及渭北台塬、南塬水土流失治理和大荔荒漠生态系统修复，构建生态安全廊道体系。实施采煤沉陷区治理和非煤矿区环境综合整治。推进节能降耗，实施重点用能企业节能行动，大力发展循环经济，促进资源节约集约利用，建设低碳社会。坚持铁腕治污降霾，实施清净水体工程和农村环境综合治理工程，让秦东大地水更清、山更绿、天更蓝。到2020年，森林覆盖率达到30%以上，单位GDP能耗累计下降16%。

　　六是持续改善民计民生，着力提升群众幸福感。加快弥补民生欠账，全面推进社会事业发展，不断为全市人民创造新福祉，让群众共享发展成果。脱贫攻坚战取得全面胜利，提前一年实现贫困县全部摘帽、非贫困县整体脱贫、贫困村全部退出、64.4万贫困人口全部脱贫。新增城镇就业25万人。健全完善城乡一体化社会保障体系，提升公共服务保障水平。实施13年免费教育，建设“千校达标”工程，推进城乡教育均衡发展，县市区全部通过省级“双高双普”验收，加快职业教育发展，打响渭南职教品牌，支持渭南师范学院建设发展。实施“健康城市”建设工程，推动医疗基础设施和诊疗服务水平持续提升，让群众在家门口享受到更好的基本医疗和公共卫生服务。巩固提升国家公共文化服务体系示范区创建成果，统筹建设各类文化体育基础设施，实施新周期全民健身计划，加强文化遗产的保护与传承，丰富群众精神文化生活。积极创新社会管理，维护社会和谐稳定，让群众生活得更加尊严、体面和舒心。

　　三、2016年的主要工作

　　今年是实施“十三五”规划的开局之年，也是决胜小康的起步之年。全市经济社会发展的主要预期目标是：生产总值增长9%左右，全社会固定资产投资增长16%，社会消费品零售总额增长13%，地方财政收入同口径增长10%，城乡居民收入分别增长10%和11%，城镇登记失业率控制在4%以内，单位GDP能耗下降3.3%。

　　着力抓好八方面工作：

　　（一）强力推动工业转型升级

　　积极适应供给侧结构性改革要求，坚持去产能与优化结构相结合，提升工业经济发展质量和效益。力争新增规模以上工业企业50户，规模以上工业增加值完成625亿元，增长10%。

　　加速产业提档升级。抢抓中省改善消费品供给、降本增效、制造业升级三个专项行动的机遇，坚持压缩和扩张并存，加大煤炭、水泥、冶金等落后产能淘汰力度，因企制宜、多措并举推动困难企业、高污染企业脱困升级、延长产业链，加快蒲城清洁能源二期、澄城烟厂技改、韩城腾龙陶瓷、潼金选厂、中垦乳业等项目建设，持续推动能源化工等主导产业向中高端发展。

　　加快培育战略性新兴产业。推进高新区3D打印基地建设，加快智能制造研发和产业化步伐，力争在雾化粉末、柔性显示面板等关键技术上实现突破。抓好高新区新能源汽车电池、德飞新能源、天臣新能源等项目建设，加快新能源汽车推广应用和充电桩规划建设。推动中节能光伏、中科新能源、中广核风力发电等项目建设，力争新增新能源电力装机300兆瓦。加大招商引资力度，紧扣“中国制造2025”，紧盯央企京企省企、东部产业转移和西安城区企业外迁，多渠道招引一批发展潜力好、带动能力强、附加值高的重大产业类项目和战略性新兴产业项目，确保招商引资到位资金650亿元。

　　提升园区发展水平。积极推进园区升级创建，支持渭南经开区创建国家级经开区、韩城经开区创建国家循环化改造示范基地，力争大荔科技园区、澄城工业园晋升省级经开区。支持渭南高新区发展“飞地”经济，拓展发展空间。创新县域园区管理机制，鼓励民营资本参与园区工业地产开发，盘活园区闲置土地和资源，加快标准厂房、配套设施和服务平台建设，着力增强园区吸纳就业、集聚人口、承载项目的能力。

　　大力推进“双创”工作。推广临渭区、高新区经验，以工业园区、商务楼宇为重点，年内创建省级“众创空间”1个、市级10个。用足用活中小微企业发展政策，加快组建“天使基金”，支持初创企业，积极培育市场主体，大力发展民营经济。引导鼓励企业走向资本市场，推动更多企业上市融资。

　　（二）扎实推进特色现代农业发展

　　做大做强特色产业。落实粮食安全行政首长责任制，新增高标准农田18万亩，发展节水灌溉10万亩，确保粮食产量稳定在210万吨以上。持续推进农业结构调整，加快苹果、酥梨、葡萄、冬枣、蔬菜、软籽石榴和生猪、奶畜等产业规模扩张和品质提升，推广种养结合模式，大力发展生态农业，力争年内新增水果10万亩、设施瓜菜4万亩以上，新建扩建标准化规模养殖场300个以上。

　　提升现代农业园区承载力。积极培育新型经营主体，推进土地向园区流转集中，加强基础设施建设，完善功能配套，推广标准化生产管理技术，加快品牌创建，增强园区的示范引领作用。年内新增3个省级、10个市级现代农业园区，园区总面积达到86万亩。

　　促进一二三产融合发展。加快渭南经开区省级农产品加工示范区和大荔、富平、澄城、白水等农产品加工集中区建设，引进培育农产品深加工龙头企业，推动食品工业强市建设，年内新增市级龙头企业20户以上。实施“互联网+现代农业”行动，大力发展休闲观光农业、乡村生态旅游等新业态，让农民分享全产业链价值收益。

　　（三）推动第三产业加速提档

　　顺应个性化、多样化消费趋势，加大优质新型服务产品的有效供给，培育消费热点，力争第三产业增加值增长9.5%。

　　推进文化旅游深度融合。坚持品牌引领，持续推进大华山旅游目的地建设，加快华山养生苑、临渭桃花源、潼关古城、渭华起义纪念地、富平国际陶艺文旅园、蒲城桥陵泰陵、白水仓颉庙、澄城尧头窑、大荔沙苑等重点项目建设，加强住宿、餐饮、停车、娱乐等配套设施建设，增强景区承载能力。大力发展红色旅游，推动洽川创建5A级景区，支持华阴、韩城创建国家级全域旅游示范市。大力开发文化演艺等特色旅游产品，打造精品线路，加强对外宣传与合作，提升渭南旅游全域化、特色化、品质化发展水平，力争接待游客人数和旅游综合收入分别增长10%以上。

　　培育壮大现代服务业。紧扣新型工业化和“互联网+”，积极发展电商、咨询、中介等新兴服务业和融资租赁、信息技术等生产性服务业。大力发展现代物流业，加快商贸物流园、铁路物流港等项目建设。年内建成5个电商孵化园，培育10户电商龙头企业，富平、大荔、澄城、韩城争创省级电子商务示范县（市）。加强金融组织体系建设，年内力争引进域外银行2家，重视发挥保险业作用，严厉打击恶意逃废债务和非法集资行为，优化金融生态环境。

　　促进消费快速增长。大力发展健康养生、家政养老等新业态，加快国家体育产业联系点城市建设，积极拓展假日经济、文化休闲等热点消费，扩大汽车、家电等大宗消费，开拓农村市场，推动消费升级增量。精心办好第26届中国厨师节，加快特色美食街区建设。抓好重点服务产业标准体系的制定和推广，推动服务业向专业化和品质化发展。年内新增限额以上贸易企业70户，社会消费品零售总额达到570亿元。

　　（四）统筹城乡协调发展

　　提高中心城市辐射带动能力。实施建设项目139个，完成投资144亿元。抓好渭东新城、西海公园、渭水生态城、人民公园建设及渭清公园提升改造，新建续建渭源街、解放路北段等13条主次干道，实施北水厂、集中供热管网、综合管廊等基础设施项目，推进海绵城市和智慧城市建设。突出加快华州区发展，推进产业布局、基础设施、公共服务等与主城区全面对接，开通城市公交，启动实施渭华快速干道。支持临渭区加快老城开发和城中村改造，鼓励高新区大力实施创新驱动战略，推进产城深度融合。加快渭河城区段景观建设，推动经开区在基础设施、产业发展等方面加速提档，增强辐射带动能力。以国家文明城市、园林城市创建为抓手，狠抓精细化管理，全面提升城市品位。

　　突出县城建设。积极推动县城周边乡镇转街办、村改社区工作，拉大框架，优化路网，加快水、电、气、热等基础设施建设。坚持差异化、特色化，突出历史文化传承，完善功能配套，大力实施“绿化亮化美化”工程，增强县城吸引力。

　　全面提升城镇综合承载能力。启动修编新一轮城市总体规划，科学确定中心城市和县城定位。完善城镇规划和村庄规划，推进富平、华阴、大荔、韩城“多规合一”，实现城乡规划全覆盖。抓好庄里镇级市试点，持续推进重点示范镇、文化旅游名镇和特色小镇建设，狠抓新型农村社区提档升级。积极推动房地产去库存，着力建设户型合理、配套完善、环境优美的高品质小区。加大棚户区改造力度，实施棚改1.89万户，农村危房改造2.35万户。扎实推进城镇基本公共服务向常住人口全覆盖，年内城镇化率达到45%。

　　（五）加快弥补基础设施短板

　　做好项目策划和资金筹措。紧扣中省政策取向，超前谋划储备一批重大基础设施项目，加大专项建设基金等争取力度，大力推广PPP模式，多渠道筹措建设资金。力争全社会固定资产投资完成2420亿元。

　　推动重大交通项目建设。加快蒲白高速、沿黄公路渭河特大桥、黄河禹门口大桥、国道310、国道342等工程建设，力促韦罗高速尽快复工，启动建设关中环线东扩项目，积极做好京昆高速改扩建、合凤高速、省道209等前期工作，新建改建县乡公路350公里，力争公路建设完成投资30亿元。持续推进高铁公交化运营，争取动车在蒲城设立停靠站。扎实推进韩城、华山支线机场和渭南至潼关、渭南至蒲城轨道交通建设等前期工作。

　　推进水电热气等基础建设。全面启动渭南水系建设，编制建设规划和方案，掀起新一轮水利建设热潮，年内完成投资15亿元。加快卤阳湖湿地生态水系建设，推进黄河小北干流和南山支流综合治理，加快二华蓄滞洪区尾留工程建设，实施二华干沟改造利用工程。推动骨干水源工程建设，年内建成抽黄供水工程，启动引清济湭和五大灌区扩灌工程前期工作。力争中心城市热电联产项目上半年开工、富平热电联产项目年内建成，建设中心城市等3个110千伏变电站，实施农网改造升级工程。扎实推进“气化渭南”，建成卤阳湖至白水、合阳至澄城等输气管道，年内县城全部实现管道供气，天然气用量突破4亿立方米。

　　（六）强力推进生态文明建设

　　狠抓节能降耗。严格落实节能减排责任制，突出抓好电力、钢铁、焦化等领域节能降耗，编制重点用能企业温室气体排放清单，开展碳排放权交易试点。严格公共机构节能考核，推广应用节能新技术、新产品，开展低碳商业、低碳城镇和低碳社区试点，大力倡导低碳生活。

　　加大环境治理力度。扎实推进“治污降霾·保卫蓝天”和新一轮渭河治理三年行动，集中开展重点流域、区域和行业污染综合整治，加快蒲电、渭化等提标改造，加强城市污水归集和污泥安全处置，加快工业园区污水处理厂建设，强化油烟、扬尘和尾气整治，做好饮用水源地保护。中心城市空气质量优良天数保持在260天以上，渭河出境断面水质稳定达到Ⅳ类。

　　致力改善生态环境。强化“生态红线”意识，坚决打击非法采石，切实抓好秦岭北麓和渭北采石区生态修复。实施“绿化秦东大地、建设美丽家园”三年行动，加快郑西、大西高铁绿色长廊建设，扩大黄渭洛河防护林规模，完成造林绿化35万亩、治理水土流失350平方公里。加强自然保护区、森林公园、湿地公园管理，加快黄河湿地晋升国家自然保护区工作。深入推进美丽乡村建设，根本改善农村环境面貌。

　　（七）以更大力度保障和改善民生

　　打好精准扶贫攻坚战。按照与城镇、园区、中心村“三靠近”原则，积极推进易地搬迁，年内搬迁1.3万户4.9万人。统筹推进贫困村水、路、电等基础设施建设，大力扶持旅游、光伏、电商等产业项目，实现产业脱贫。建立多层次、全覆盖的贫困家庭在校生助学体系，强化教育脱贫。实行农村低保标准与脱贫标准“两线合一”，全面开展重特大疾病救助，落实社保兜底脱贫。年内确保21万人脱贫、230个村退出贫困村。

　　做好就业和社会保障工作。突出抓好高校毕业生、农民工、“去产能”分流人员等群体就业创业服务，全年新增城镇就业5万人，农村劳动力转移就业80万人。探索推动城乡居民基本医疗保险制度整合，完善大病保险制度，扩大住房公积金制度受益面。做好失地农民、个体从业人员和农民工等群体的社保工作。加快社会救助体系、养老设施建设，支持发展多种形式的养老服务机构。

　　积极发展社会事业。推进“千校达标”工程和“九项示范校”创建，新建改建公办幼儿园81所，创建标准化学校150所，中心城市新建4所中小学校，加大县城学校建设力度。力促潼关、华阴、白水通过国家义务教育均衡县验收，澄城、华州等县市区通过省级“双高双普”验收。实施高中阶段三年免费教育，促进普通高中特色发展，加快中职资源整合，鼓励发展民办教育。全面推行分级诊疗，建立全科医生制度，推进县镇村医疗一体化。全面落实一对夫妇可生育两个孩子政策。实施渭河沿岸全民健身长廊工程，推进县级健身活动中心和镇村文化体育惠民工程建设，确保公共文化服务体系示范区创建通过国家验收。办好全国青少年“未来之星”阳光体育大会及“丝绸之路”国际青年足球赛、国际马拉松赛、自行车赛等重大赛事，保障群众基本文体需求。

　　着力维护和谐稳定。坚持以“零容忍”的态度抓好安全生产，强化重点行业安全监管，严格责任追究，坚决杜绝重特大事故发生。深化信访制度改革，健全多元化矛盾纠纷调解和重大决策社会稳定风险评估机制，畅通群众诉求表达渠道，妥善化解各种不稳定因素。强化食品药品监管，保障群众饮食用药安全。做好新形势下民族工作，依法管理宗教事务。扎实推进平安渭南建设，严厉打击各类刑事犯罪，确保全市社会大局稳定。

　　持续推进双拥共建。一如既往地支持驻渭部队建设，拥护支持军队改革，深入开展国防教育，加快推进军民融合，健全国防动员体系，搞好预备役部队和民兵建设，推进国家双拥模范城创建，不断开创双拥共建工作新局面。

　　（八）持续推进改革创新

　　深化重点领域改革。全面做好市县政府机构改革和镇村综合改革后续工作，加快事业单位分类改革。持续推进简政放权，落实“权力清单”、建立“责任清单”，提升行政审批效率，规范中介服务行为。加强市行政服务中心标准化建设，年内建成县、镇、村三级政务服务体系，积极推进网上办事。加快国家中小城市综合改革试点，支持韩城计划单列市试点建设。推进财税体制改革，实施零基预算管理，加强财政资金统筹使用，提升财政资金管理绩效。深化国企改革，着力推动中介机构与主管部门脱钩，稳妥清退“僵尸”企业，开展国有资本投资运营试点，促进混合所有制经济发展。深化农村改革，加强“三资”管理，扎实做好农村土地确权登记颁证工作。

　　推动创新型渭南建设。实施科技统筹创新工程，加快建设以企业为主体的创新体系，提升市科技资源统筹中心服务能力，大力引进创新型人才，着力提高科技创新能力。年内新建市级工程技术研究中心和技术创新战略联盟10个、科技创新服务平台10个，技术合同成交总额达到2亿元，专利授权量达到500件。

　　扩大对外开放。推动晋陕豫黄河金三角、西渭、富阎基础设施互联互通和产业协同发展，争取与西安在口岸、综合保税区等开放政策上互认共享。充分发挥渭南海关作用，推广“铁海联运”通关模式，支持渭南高新区发展外向型经济，促进外贸稳定增长，力争全年进出口总额实现2亿美元。

　　四、切实加强政府自身建设

　　新常态、新形势、新目标，要求我们必须转变职能、依法行政、创新管理，全面提高政府治理现代化水平。

　　依法履职尽责。深入推进法治政府建设，健全依法决策机制，坚持重大决策合法性审查，推进行政机关法律顾问全覆盖，不断把政府工作纳入法治化轨道。扎实开展“七五”普法，巩固依法行政示范市创建成果。依法接受市人大及其常委会的监督，主动接受市政协的民主监督，广泛听取民主党派、工商联、无党派人士、各人民团体的意见，重视司法、舆论和社会监督。加大政务公开力度，坚持开展百姓问政、向人民报告、邀请公民进政府等活动，努力打造运行规范、公开透明的阳光政府。

　　持续改进作风。深化“三严三实”专题教育成果，持续推动政府职能转变，优化提升服务质量。坚持深入基层、深入群众调查研究，从群众中不断汲取智慧和力量。建立合理容错机制，把干部在推进改革发展中因缺乏经验而出现的无意过失和错误失误，与令行不止、明知故犯、以权谋私等违纪违法行为区分开来，鼓励干事，激励探索，最大限度地激发广大干部的创业创新热情。完善落实推进机制，加强绩效管理，健全考评机制，加大问责问效，严肃整治不作为、慢作为和乱作为，努力打造勤政精干、务实为民的效能政府。

　　加强廉政建设。从严落实党风廉政建设“一岗双责”，严格落实述职述廉、诫勉谈话、质询问责等制度，扎实推进惩治和预防腐败体系建设。前移审计、监察监督关口，强化政府采购、工程招投标、公共资源交易等领域监管，从源头上预防腐败。大力倡俭治奢，坚持政府所有收支一律纳入预算管理，所有公共资金一律接受审计监督，确保财政供养人员只减不增，会议、文件持续减少，“三公”经费支出不断下降，努力打造纪律严明、风清气正的廉洁政府。

　　各位代表，渭南发展已站在新的起点，追赶超越的蓝图催人奋进，决胜小康的号角已经吹响！让我们在市委的坚强领导下，坚持低调务实不张扬，埋头苦干，奋力拼搏，为建设富裕、和谐、文明、绿色、开放新渭南而努力奋斗！

　　名词解释

　　〔1〕引石济白：工程通过输水管网接引石堡川水库水自流至白水县城，以解决白水县城、园区、沿线群众及部分农田灌溉缺水问题。

　　〔2〕中心城市“一河两岸、五大组团”：根据《渭南市城市总体规划》，中心城市主城区围绕将渭河建成重要的生态景观廊道，形成南北两岸共同发展的空间格局；五大组团指中心区组团、高新区组团、经开区组团、华州区组团和卤阳湖组团。

　　〔3〕“两纵四横”高速枢纽网络：两纵为榆蓝、榆商高速公路；四横为合（阳）凤（翔）、连霍、京昆及渭（南）运（城）高速公路。

　　〔4〕“两纵两横”一级公路主骨架：两纵为省道209白水至渭南段、国道242澄城至大荔段；两横为国道108富平至韩城段、国道310华阴至渭南段。

　　〔5〕“一核三带五板块”：“一核”即中心城市，“三带”即富平——韩城城乡统筹示范带、渭南——潼关城乡统筹示范带、黄河西岸城乡统筹示范带，“五板块”即中心城市高端制造和现代服务业板块、韩城新型工业和循环经济板块、富蒲航空和卤阳湖湿地板块、大华山山水文化旅游板块、蒲白澄合载能工业和绿色生态板块。

　　〔6〕引清济湭：规划在西安市蓝田县清峪河新建清峪水库，通过输水建筑物将清峪河水引至湭河上游支流稠水河，年可向湭河水库调水1692万立方米。
